# 

**Published:** 1967-01

**Authors:** 


### BRISTOL MEDICO-CHIRURGICAL JOURNAL

(*Incorporating the Medical Journal of the South-West*)

Established 1883

Vol. 82 (i) January, 1967 No. 303

### *Contents*

Page

ON LISTENING TO THE NECK ........................ *T. W. Lloyd* 1

DIPYRIMADOLE & POST-OPERATIVE HYPERCOAGULABILITY ........................ *U. Jayaswal* 8

AN APPROACH TO THE ANALYSIS OF MENORRHAGIA *P. F. Fairbrother* 11

CARCINOMA OF THE LUNG WITH CUSHING'S SYNDROME: A Clinico-Pathological Conference 15

*PUBLISHED QUARTERLY*

*ANNUAL SUBSCRIPTION* **16/-** *POST FREE*

### BRISTOL MEDICO-CHIRURGICAL SOCIETY

President 1966-67 : Dr. S. F. Marwood.

President-elect : Professor T. F. Hewer.

Honorary Secretary : Dr. A. T. M. Roberts, 8 Harley Place, Bristol 8.

Honorary Treasurer : Dr. J. Macrae, Ham Green Hospital, Pill.

The Society was founded in 1874. In 1883 publication of this Journal began, and has continued quarterly since then. During the years 1949 to 1962 it appeared under the title of " The Medical Journal of the South West ".

The Society founded a medical library in 1890, which in 1892 was combined with that of the Bristol Medical School. This became the University of Bristol Medical Library in 1925, and an agreement in that year confirmed the privilege of Members to use the University Medical Library.

### THE BRISTOL MEDICO-CHIRURGICAL JOURNAL

*Editorial Committee*

Dr. N. J. Brown, Southmead Hospital, Bristol. *Joint Editor.*

Dr. A. B. Raper, Department of Pathology, Bristol Royal Infirmary. *Joint Editor.*

Dr. J. Macrae, Ham Green Hospital, Pill, Bristol. *Honorary Treasurer.*

Dr. R. C. Wofinden, Central Clinic, Bristol.

Mr. A. E. S. Roberts, Medical School Library, University Walk, Bristol 8. *Secretary.*

### NOTICE TO CONTRIBUTORS

The Journal is especially concerned to provide a place for the recording of medical thought, interests, and practice in and around Bristol. It therefore welcomes articles that, as well as being of immediate interest to members, will document the local and contemporary medical scene for our successors.

Original articles are invited provided that they have not been published elsewhere. References in the text should be by author's name, with year of publication in parenthesis; they should be listed at the end in alphabetical order. Illustrations should be supplied ready for reproduction. Line drawings should be in indian ink on firm white paper or board. The author will be informed if it is found necessary to ask him to pay for the making of blocks. The Editorial Committee reserves the right to make literary corrections.

The following contributions will be especially welcome : notes of forthcoming events, meetings held, degrees conferred, staff appointments, honours and public appointments, and other local news.

### BRISTOL MEDICO-CHIRURGICAL SOCIETY, 1965-1966

President : Mr. G. M. FitzGibbon

The following meetings were held during the session :—

| | |
|---|---|
| 13th Oct. 1965 | Presidential Address, " Pictorial Art in Medicine ". |
| 10th Nov. 1965 | Clinical Meeting, Bristol Royal Infirmary. |
| 8th Dec. 1965 | Mr. D. C. Bodenham, " Tumours of the Skin ". |
| 12th Jan. 1966 | Dr. D. W. Pugh, " Recent Aspects of Diabetes ". |
| 9th Feb. 1966 | Sir Theodore Fox, " Population and People ". |
| 9th Mar. 1966 | Prof. A. G. Riddell, " The Evolution of Liver Surgery ". |
| 13th Apl. 1966 | Sqdr. Ldr. E. M. B. Smith, " Medical Problems of Spac[illegible] Travel ". |
| 11th May 1966 | Dr. J. M. Naish, " The Advance of Gastroenterology ". |

For the first time in the history of the Society, it was found necessary t[illegible] raise the subscription; the new rate of two instead of one and a half guinea[illegible] became operative from the beginning of the session.

27 new members were proposed and accepted, and at the end of the yea[illegible] the membership stood at 555.

